# An improved modeling approach to investigate biases in human random number generation

**DOI:** 10.1371/journal.pone.0324870

**Published:** 2025-05-29

**Authors:** Tim Angelike, Jochen Musch

**Affiliations:** Department of Psychological Assessment and Differential Psychology, Institute of Experimental Psychology, Heinrich Heine University Düsseldorf, Düsseldorf, Germany; NED University of Engineering and Technology, PAKISTAN

## Abstract

Recently, a novel computational model was proposed to investigate the processes and biases involved in human random number generation (RNG). This two-parameter model includes a repetition parameter and a side-switching parameter representing influences of the immediately preceding number on the choice of the next number. We propose two changes to the model. First, we replace the side-switching parameter with a more general and less task-dependent distance parameter, which accounts for the tendency to select subsequent numbers that tend to be either closer to or further away from the previous number on the selected response pad. Second, we extend the computational model by adding a third parameter to account for the human tendency to select subsequent numbers with greater probability the longer the respective number has not been previously selected, following the pattern of the well-known gambler’s fallacy. This new “cycling” parameter takes into account the most recent and all previous selections. The generalized distance parameter, and particularly the new cycling parameter, improved the fit of the model to human-generated sequences and the rate of successful predictions of the next choice from 14.09% to 26.48%, significantly exceeding the expected chance value of 1/9 = 11.1%. Model-driven simulations also showed that the extended three-parameter model could better account for systematic patterns that can be observed in human RNG tasks. The improved model could be useful in many contexts where human biases in RNG tasks are analyzed.

## Introduction

Researchers typically investigate the ability to generate random-like sequences of numbers using random number generation (RNG) tasks in which participants are asked to generate a random sequence of numbers [1–3]. The general consensus from these studies is that humans encounter significant challenges when attempting to generate truly random number sequences, and exhibit various dependencies between subsequent responses when generating a random-like series of numbers [4,5]. For example, people tend to avoid immediate repetitions of numbers and often exhibit a tendency to repeat specific pairs of numbers.

The present study tries to gain a better understanding of the cognitive processes underlying human deficiencies in random number generation. It builds on the work of Yousif et al. [6] who introduced a computational approach to account for potential biases, involving two parameters that model human behavior in RNG tasks: a repetition parameter expressing the tendency to generate too few or too many repetitions and a side-switching parameter indicating the propensity to switch between higher and lower numbers. In the present contribution, we propose two modifications to Yousif et al.’s original model. First, we replace the somewhat domain-specific side-switching parameter with a more generalized distance parameter that characterizes the decision to choose a number as a function of its distance from the previously selected number. Second, we expand the computational model with a third parameter that accounts for the human tendency to cycle too rapidly through all possible numbers in a sequence. The cycling parameter constitutes the primary contribution of the present paper. The previous models solely incorporated parameters that represent simple first-order dependencies between adjacent numbers in a sequence. In contrast, the cycling parameter that is at the heart of the new model describes the generation of random sequences on a more global scale by accounting for more complex dependencies. To this end, the complete prior choice history is considered when predicting subsequent choices in RNG tasks.

The ability to generate sequences of numbers that appear random is a subject of frequent investigation through the RNG task [1,3,7]. In this task, participants are asked to generate a sequence of numbers within a discrete interval, typically ranging from 1 to 9, aiming for maximum randomness [8–10]. A common observation in this task is that humans often struggle to produce random-like sequences and exhibit serial correlations between consecutive responses [4,5,10]. One of the most noticeable errors individuals make when attempting to generate random number sequences is a significant reduction in the frequency of immediate repetitions of a number, as compared to what would be expected by chance in a truly random sequence [1,11]. The avoidance of such repetitions is so pronounced that their occurrence is often exceedingly rare. Studies have demonstrated that this bias remains consistent in humans, irrespective of variations in RNG task parameters, including production speed, the set of possible numbers for sequence generation, task modality, and cognitive load [12,13]. A potential explanation for this bias may be that people generally perceive repeated numbers as less random than alternating ones [14,15]. Additionally, humans exhibit a systematic tendency to repeat specific numerical patterns [1,3,10,12]. One example of such a systematic pattern is the inclination to switch from the lower (1,2,3,4) to the higher half (6,7,8,9) of numbers (e.g., 3–8, or vice versa, 8–3) [6].

The challenge of generating random numbers may arise from the involvement of multiple cognitive processes required to complete this task. There is evidence that RNG tasks require different cognitive functions, including working memory, monitoring, and inhibitory ability [11,13,16,17]. Notably, performance in RNG tasks is compromised in individuals with neurological and psychiatric disorders [18–20]. Schizophrenic patients, for example, often exhibit a strong inclination to repeat specific patterns, such as adjacent pairs [2]. The relevance of understanding impairments in random number generation to the comprehension of clinical disorders underscores the importance of further exploring the human capacity to generate random numbers and the processes involved.

Recently, Yousif et al. [6] proposed a cognitive model aimed at elucidating biases exhibited by humans when generating sequences of random numbers. Initially designed for RNG tasks involving the generation of sequences of numbers ranging from 1 to 9, the model can be generalized to any RNG task, regardless of the allowed number range. Yousif et al.’s model introduces two parameters representing biases in RNG tasks: a repetition parameter and a side-switching parameter. The repetition parameter, denoted as ϵ, captures the systematic tendency to produce too few or too many repetitions in a sequence. Typically, humans exhibit a tendency to show too few repetitions in RNG tasks [1,12]. The side-switching parameter, denoted as η, reflects the inclination to switch from lower to higher numbers and vice versa in consecutive responses. Yousif et al. [6] demonstrated the presence of side-switching behavior in human-generated sequences. Combining these two biases, the probability $\sigma (z{)}_{i}\;$of choosing the *i*-th number in the vector of all *K* possible numbers ranging from 1 to *K* is expressed using the following formula:


$$\sigma (z{)}_{i}=\frac{e^{\epsilon \cdot r_{i}+\eta \cdot s_{i}}}{\sum_{j=1}^{K}e^{\epsilon \cdot r_{j}+\eta \cdot s_{j}}}$$ (1)

In this equation, ϵ represents the repetition parameter and η represents the side-switching parameter. The repetition parameter influences the probability of choosing a number based on whether it is a repetition of the previous number. The model accounts for this tendency by multiplying the repetition parameter by the value *r*_*i*_ in the vector *r* (length *K*), which is coded as 1 at position *i* if the previous number in the sequence was the same number and 0 otherwise. This ensures that the repetition parameter only directly affects the probability of the number that would be a repetition of the previous number. Positive values in the repetition parameter indicate a tendency to repeat a number. Negative values indicate a tendency to avoid repetitions (fear of repetition). A parameter value of 0 indicates the absence of any bias.

The side-switching parameter (η) is associated with the tendency to switch between lower and higher numbers. For this parameter, the possible numbers from 1 to 9 are divided into two sides: lower numbers in the range from 1 to 4 and higher numbers in the range from 6 to 9 (each side consisting of four numbers). The number 5 is defined as the separator between the two sides and is considered to be neither a lower nor a higher number. The value of the side-switching parameter is multiplied by the value *s*_*i*_ of the vector *s* (length *K*), which is coded as 1 if the previous response was from the other side (e.g., if a 3 precedes a 9) and -1 if the previous response was from the same side (e.g., if a 3 precedes a 4). The value of the vector entry representing the number 5 is always coded as 0 as this number does not belong to either side. The entire vector is coded as 0 if the previously generated number was a 5, as a side-switch is by definition not possible in this case. Positive values in the side-switching parameter indicate a tendency to switch from lower to higher and from higher to lower numbers (e.g., from 2 to 8 or from 9 to 3). Negative values indicate the tendency to stay on the same side (e.g., a 4 after a 3 or a 7 after a 9). Again, a value of 0 indicates no bias at all.

The numerator on the right-hand side of Equation 1 is the weight associated with the selection of the *i*-th number, which is the sum of the effects of the repetition and the side-switching parameter. To convert this weight for a number into a probability, it is divided by the sum of the weights of all the possible numbers that can produced (the denominator). This ensures that all probabilities $\sigma (z{)}_{i}$ lie between 0 and 1 and sum to 1.

Using modeling approaches such as that by Yousif et al. [6], it is possible to formalize the biases that humans show when trying to generate random sequences as model parameters. In this way, Yousif et al. were able to directly estimate the putative latent variables behind RNG performance: in this case, the tendency to make or avoid repetitions, quantified by the repetition parameter, and the tendency to either switch from lower to higher numbers and vice versa, or to prefer to stay on one side of the number range, quantified by the side-switching parameter. Their model-based approach also allowed them to determine the fit of their model, which is useful for comparing models and testing whether or not to add a parameter. Finally, models of human number generation can be used to simulate sequences of numbers that should resemble human-generated sequences which Yousif et al. successfully used to test model predictions. The model by Yousif et al. [6] was also applied recently to investigate the temporal stability of the response patterns underlying RNG tasks in Boger et al. [21].

Our first goal was to make the original by Yousif et al. [6] model less dependent on the format of the RNG task. Specifically, we replaced the side-switching parameter with a distance parameter that describes the selection of a number in an RNG task as a function of the distance between the previously selected number and the currently selected number. This means that it is possible to describe systematic behavior in an RNG task where numbers close to the previously selected number (e.g., pairs such as 7–6) are generated with either increased or decreased probability. This parameter is conceptually related to the idea of the side-switching parameter, where numbers from two different sides (lower or higher side) are roughly related to larger distances and numbers from the same side to smaller distances. It was included to be able to account for the findings of Towse [12] who demonstrated a tendency of human participants to generate adjacent pairs of numbers, which would result in negative values of the distance parameter reflecting higher probabilities for numbers that have a small distance to the previously generated number.

The new distance parameter has the advantage of being less dependent on the specifics of the RNG task, as it can be tailored to any possible order of the numbers that can be used to generate the sequence. This allows task-specific features to be included in the modeling process. For example, in the second experiment by Yousif et al. [6], participants generated random numbers by clicking on a horizontal line. For this one-dimensional response format, the distance could be computed as the numerical difference between the numbers (e.g., the distance in the response pairs 4–7 and 7–4 would be 3). This procedure for calculating the distances can also be used for oral production formats of RNG tasks, which are commonly used as well [1,2,10,12]. One possible prediction for an oral production format would be that the distances between numbers are best represented by their numerical distance, which is equivalent to representing the numbers on a horizontal line. However, numbers in an RNG task can also be presented in a 3x3 matrix [9,22], which could be accounted for by calculating the distances between numbers in the two-dimensional space of possible numbers. A simple dichotomization into lower and higher numbers is necessarily somewhat arbitrary as there are several ways on how to dichotomize a two-dimensional matrix of numbers (e.g., lower vs. upper or left vs. right side). Replacing the side-switching parameter with the distance parameter in the model, we get


$$\sigma (z{)}_{i}=\frac{e^{\epsilon \cdot r_{i}+\delta \cdot d_{i}}}{\sum_{j=1}^{K}e^{\epsilon \cdot r_{j}+\delta \cdot d_{j}}}\;.$$ (2)

The distance parameter δ is multiplied by the distance *d*_*i*_ in the vector *d* of length *K,* which encodes the distance of a number from the previously generated number. Positive values of the distance parameter lead to higher probabilities for numbers that have a large distance to the previously generated number, while negative values of the distance parameter lead to higher probabilities for numbers that have a smaller distance to the previously generated number. A parameter value of 0 indicates the absence of any systematic behavior.

However, previous research has found another peculiarity that is not accounted for in the original model by Yousif et al. [6]. Humans have been observed to exhibit a tendency to cycle too quickly through all available numbers in an RNG task [1–3,9]. Cycling describes the behavior whereby people tend to generate numbers with a higher probability the longer they have not been used in a sequence. For example, if the number 6 has not been generated recently, it is more likely to be generated next in the sequence than a number that has already been generated recently. This tendency to cycle will also lead to faster completion of the full set of numbers on average, as an attempt is made to use all possible numbers as evenly as possible. This bias is related to the concept of the gambler’s fallacy [23], where people mistakenly believe that a number that has not come up for a long time is likely to come up next, even though the probability of each number remains the same in each round. To account for this tendency, we propose to extend the modified version of the two-parameter model by adding a third parameter reflecting a cycling bias. A major advantage of an additional cycling parameter over the repetition, side-switching, and distance parameters is that it uses the entire prior choice history, not just the immediately preceding choice, to describe the subsequent choice. Therefore, the cycling parameter describes the generation of numbers in an RNG task on a more global scale than the two parameters of the original model. In this way, it should be possible to capture systematic patterns in human-generated sequences, such as repetitions of numbers separated by a gap of several other numbers, thereby going beyond simple first-order dependencies.

In the following, we will refer to the new cycling parameter as β. Positive values of this parameter indicate a tendency to cycle through all available numbers too quickly. Negative values indicate that the probability of selecting a number decreases the longer it has not been selected. The value 0 indicates the absence of any bias. Adding the third parameter to the model in Equation 2, we get


$$\sigma (z{)}_{i}=\frac{e^{\epsilon \cdot r_{i}+\delta \cdot d_{i}+\beta \cdot g_{i}}}{\sum_{j=1}^{K}e^{\epsilon \cdot r_{j}+\delta \cdot d_{j}+\beta \cdot g_{j}}}\;.$$ (3)

Here, *g* is the gap vector of length *K,* which codes at each position *i* how long a number has not been used. Each time a number is selected, its gap value is set to 1, and the gap value of every other number is incremented by one. At the beginning of a sequence, each gap value is initialized to 1. The model assumes that the effect of the cycling parameter increases linearly with the number of times a number is not selected. However, assuming a linear trend would consider a gap increase from 2 to 4 as equivalent to a gap increase from 12 to 14, even though the latter would be a much smaller increase on a relative scale. We therefore also consider a modification of the model that scales the gap to the last occurrence of a number on a logarithmic scale. This expresses the idea that once the distance to the last occurrence of a number is already large, the effect of increasing the distance further becomes smaller and eventually reaches an asymptote. This way, we get


$$\sigma (z{)}_{i}=\frac{e^{\epsilon \cdot r_{i}+\delta \cdot d_{i}+\beta \cdot \log_{2}g_{i}}}{\sum_{j=1}^{K}e^{\epsilon \cdot r_{j}+\delta \cdot d_{j}+\beta \cdot \log_{2}g_{j}}}\;.$$ (4)

The advantage of adding a cycling parameter is that it reflects a well-documented human bias in RNG tasks [1,2,24] and allows more complex dependencies to be uncovered. The repetition, side-switching, and distance parameters only consider influences of the immediately preceding number on the following number, whereas the cycling parameter describes the choice of the following number as a result of the complete prior history of choices. To test which model best describes the human-generated sequences produced in RNG tasks, we used the data from an RNG task by Angelike and Musch [24].

### Hypotheses

1. We hypothesized that the new distance and cycling parameters would improve model fit and allow us to better account for observed human behavior in an RNG task. This expectation was based on the finding that humans have been observed to prefer to generate pairs of adjacent numbers [1,12] and to show a tendency to cycle through all available numbers too quickly when attempting to generate random sequences [1,2].
2. We expected that the model would also to be able to account for systematic behavior that humans show in RNG tasks. This hypothesis is based on the findings by Yousif et al. [6], who showed that simulated sequences of numbers based on the participants’ fitted parameter values exhibited systematic features that were not explicitly modeled. For example, they calculated the number of direction switches between ascending and descending subsequences of numbers. This analysis showed that the model-generated sequences had similar characteristics to the human-generated sequences in terms of this metric. We extended this approach by utilizing a more extensive range of randomness measures to assess systematic features in sequences to determine the effectiveness of our enhanced model in capturing higher-order properties compared to the two-parameter model without a cycling parameter. We used various measures that were identified as being highly sensitive to systematic patterns observed in human-generated sequences in a recent study [24].

## Methods

**Fig 1:**
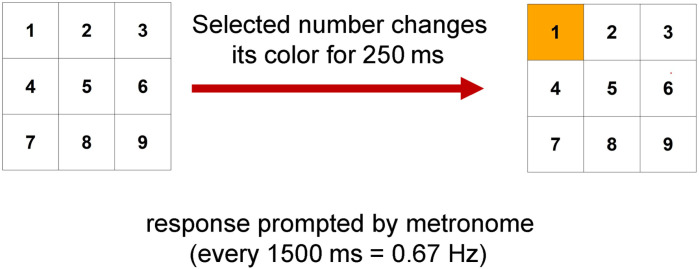
The RNG task.

The data for our analyses was obtained from Angelike and Musch [24]. In their study, participants completed an RNG task through an online platform, whereby 200 consecutive numbers were generated between the range of 1–9 every 1.5 seconds, accompanied by a metronome sound. Participants selected a number by clicking on it in a 3x3 grid, resembling a telephone or ATM keypad. The first row consisted of numbers 1–3, the second row of numbers 4–6, and the third row of numbers 7–9 (see Fig 1 for an illustration). Prior to the experiment, participants were instructed about the concept of randomness. The sample comprised 830 participants (405 men, 424 women, one non-binary).

The study was carried out in accordance with the revised Declaration of Helsinki [25] and the ethical guidelines of the German Association of Psychologists and the German Psychological Society [26]. Participants were informed about the general purpose of the study, their rights, and the intended use of their data in order to obtain their written informed consent. Information on the content of the study (random number generation), the strict anonymization of all personal data, and the exclusive use of the collected data for research purposes was provided on the first page of the study. Participants could only continue with the study if they agreed to these terms. Participation was voluntary and not associated with any risk of physical or mental harm or discomfort beyond participants’ everyday experiences. Therefore, ethics committee approval was not required according to the “Ethical Research Principles and Test Methods in the Social and Economic Sciences” formulated by the Ethics Research Working Group of the German Data Forum [27] and the “Ethical recommendations of the German Psychological Society for researchers and ethics committees” [28]. The dataset was completely anonymized and can be accessed through the following link https://doi.org/10.3758/s13428-024-02456-7. The data were downloaded on January 9, 2025.

## Results

**Fig 2:**
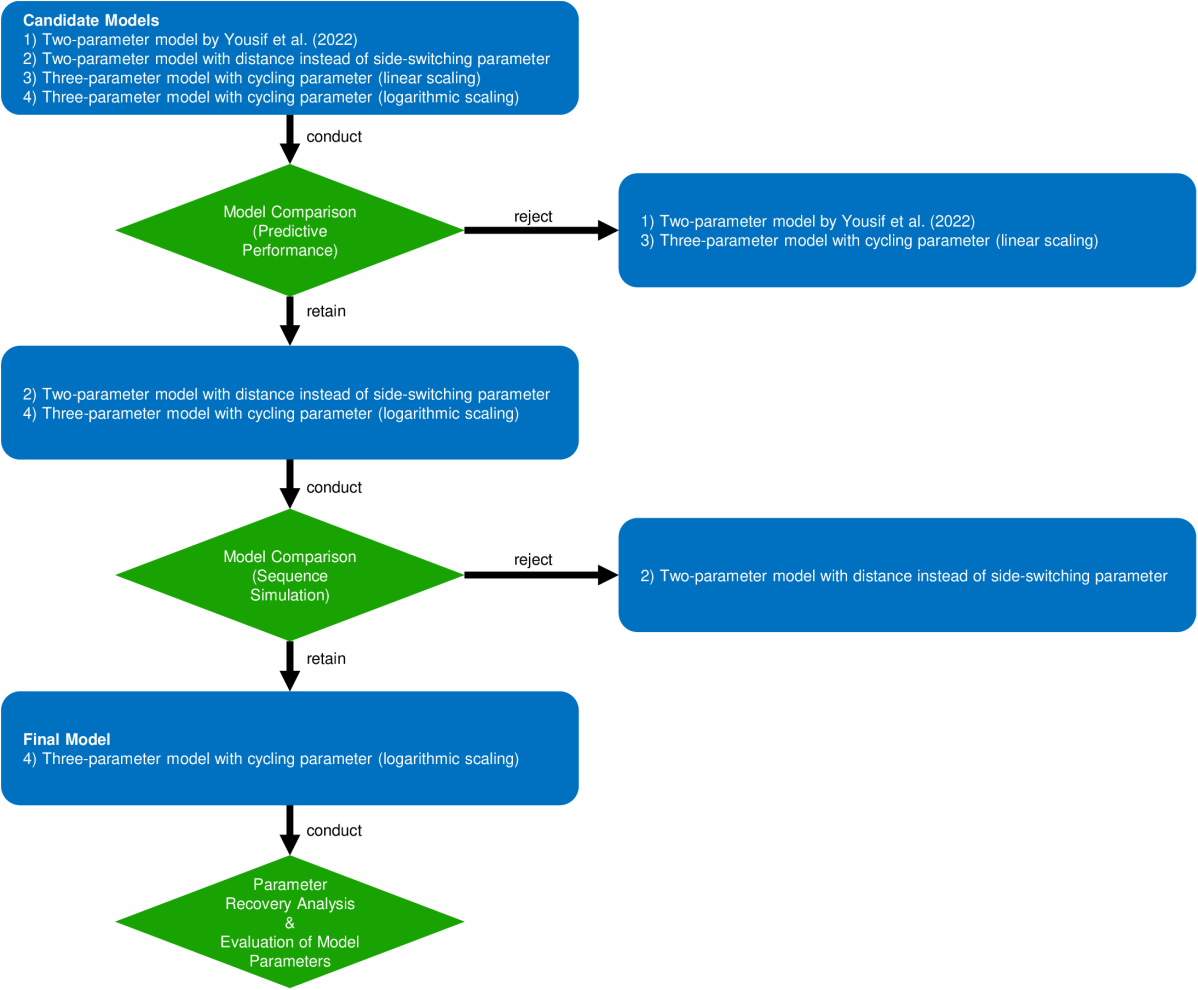
Flow chart of all steps conducted in the analyses. Note. Predictive performance is measured as the % hit rate when predicting the subsequent number in a sequence (with a chance base rate of 1/9 = 11.1%). In the second step of the analysis, the extent to which model-based simulated number sequences could approximate higher-order properties of human-generated sequences was assessed using standard measures of randomness. In the third step, the efficacy of the best performing model in reliably recovering the true parameter values was examined and the distribution of individual parameter estimates was determined.

We conducted all statistical analyses using the R programming language R 4.4.2 [29]. Additional software packages employed for analysis were rstan 2.32.6 [30], randseqR 0.1.0 [31], randfindR 0.1.0 [32], ggplot2 3.5.1 [33], ggdist 3.3.2 [34], gghalves 0.1.4 [35], ggpubr 0.6.0 [36], patchwork 1.3.0 [37], papaja 0.1.3 [38], BayesFactor 0.9.12–4.7 [39], and effsize 0.8.1 [40]. Additionally, we used the STAN programming language to obtain model estimates based on Bayesian hierarchical modeling, employing Hamiltonian Markov Chain Monte Carlo simulations using the No-U-Turn sampler [41]. The code used for all analyses can be found at https://osf.io/sygdu/. All models presented in the results section were estimated on eight separate chains with 10,000 iterations each to obtain a measure of model convergence. We discarded the first 5,000 iterations as warm-up to prevent potential convergence issues during initialization. Parameter estimates were initialized with random values in the range -2 to +2 (the default of rstan). A graphical summary of all analyses conducted in this paper can be found in Fig 2.

### Candidate models

We compared a total of four models: the original two-parameter model by Yousif et al. (Equation 1 [6]), the two-parameter model with a distance instead of a side-switching parameter (Equation 2), where the distance between numbers was computed as the Euclidean distance between the numbers in the 3x3 matrix of the RNG task used in the experiment by Angelike and Musch [24], the three-parameter model adding a cycling parameter with a linear scaling of the gap to the last occurrence of a number (Equation 3), and the three-parameter model with a cycling parameter based on a logarithmic scaling of the gap (Equation 4).

All parameters within a model were estimated jointly in a hierarchical framework. Individual parameters for each participant regarding a bias were assumed to follow a normal distribution (the prior). For example, the original two-parameter model by Yousif et al. [6] consists of the repetition (ϵ) and side-switching parameter (η), where individual parameters were assumed to follow a normal distribution with mean μ and standard deviation σ (the hyperparameters):


$$\epsilon_{n}\sim \;N\left(\mu_{\epsilon },\;\sigma_{\epsilon }^{2}\right)$$ (5)

$$\eta_{n}\sim \;N\left(\mu_{\eta },\;\sigma_{\eta }^{2}\right)$$ (6)

Here, the subscript n ranges from 1 to *N* (the sample size), identifying each participant. The subscripts for the means μ and variances $\sigma^{2}$ indicate that each parameter has its own mean and variance. Thus, we estimated both the individual parameters for each bias and their mean and variance to obtain parameter estimates at the group level, as can be seen in Equations 5 and 6. This approach was employed in estimating all four models outlined in the Equations 1–4.

The hyperparameters representing the means of the parameters were drawn from a uniform distribution ranging between −10 and 10. This cutoff was chosen as a simulation of a model-generated sequence of length 100,000 with extreme parameter values of -10 or +10 in the repetition parameter led to extreme response behavior comparable to that of participants who did not seriously participate in the task: The proportion of repetitions for a repetition parameter of -10 was 0 and 0.9997 for a repetition parameter of 10. Similar results were obtained when using these extreme parameter values for the other parameters. An uninformative prior was chosen for the hyperparameters of the means, given the absence of any prior information on parameter distribution. For the same reason, we specified a uniform prior ranging from 0 to 10 for the standard deviations of the normal distributions (square root of the variances). For all hyperparameters of the four models, the convergence diagnostic $\hat{R}$ was 1.00, indicating convergence of model estimates across all chains.

### Model selection

To assess the model’s adequacy, we calculated the average probability of predicting upcoming numbers in a sequence, starting with the second number and using the fitted parameter values from the posterior distribution. This was done separately for each participant and each model. If the model assigned the highest probability to the number that was actually selected, this number was considered a correct prediction (hit) and assigned a value of 1; otherwise, it was considered a miss and assigned a value of 0. If there were multiple numbers with the highest probability and one of them was selected, the model’s uncertainty in prediction was equally distributed among the candidate numbers that were assigned the highest probability. For example, if the model allocated an equal highest probability (25% each) of all possible numbers for the next selection to the numbers 6 and 8 and one of these numbers was picked, then the predictive accuracy for that choice was calculated to be 0.5. This corresponds to one divided by the number of alternatives that were assigned the highest probability. In this example, the model successfully predicted that either a 6 or an 8 would be chosen next, but showed no preference between these two alternatives. Finally, we calculated the average accuracy of all predictions (either 0, 1 or 1/*n*, in the event of a tie among *n* numbers for the highest probability) for each participant and for each model given the posterior distribution of the parameters. In this way, we assessed the models’ ability to forecast the subsequent response using all previously selected numbers. An overview regarding all models and their predictive performance can be seen in Table 1.

**Table 1:** Parameters and predictive performance of all models.

Parameter	Yousif et al. (2022)	Two-parameter model (distance instead of side-switching parameter)	Three-parameter model (linear scaling)	Three-parameter model (logarithmic scaling)
side-switching (η)	✔			
repetition (ϵ)	✔	✔	✔	✔
distance (δ)		✔	✔	✔
cycling (β)			✔	✔
Predictive Performance				
Mean	14.09%	15.88%	26.14%	26.48%
*SD*	4.42%	5.56%	9.29%	9.31%

Note. Predictive performance is measured as the % hit rate when predicting the subsequent number in a sequence (with a chance base rate of 1/9 = 11.1%).

First, we compared Yousif et al.’s original two-parameter model [6] with our modified version, which uses a distance instead of the side-switching parameter. The mean predictive accuracy for the modified two-parameter model (*M* = 15.88%, *SD* = 5.56%) exceeded that of the original two-parameter model (*M* = 14.09%, *SD* = 4.42%) for 70.60% of the participants. A paired sample *t*-test confirmed that there was a rise in predictive performance resulting from substituting the side-switching with the distance parameter, *t*(829) = 16.79, *p* < .001, *d* = 0.34, $\log_{10}BF=\;51.09$. Cohen’s *d* for dependent measures was calculated according to Borenstein et al. [42].

It may seem surprising that both two-parameter models can only achieve a predictive accuracy marginally exceeding the chance level of 1/9 when anticipating the following number in a sequence. However, this outcome is explicable in light of the simplicity of the two-parameter models. The repetition, side switching, and distance parameters merely reflect simple first-order dependencies. Therefore, in cases where the repetition parameter has a strong negative influence, the model can ascertain that participants are inclined to eschew direct repetitions, but cannot determine the specific number that they will select next. Even in the best scenario, the inclusion of this parameter alone can therefore only increase predictive accuracy from 1/9–1/8, assuming that there are no repetitions present.

**Fig 3:**
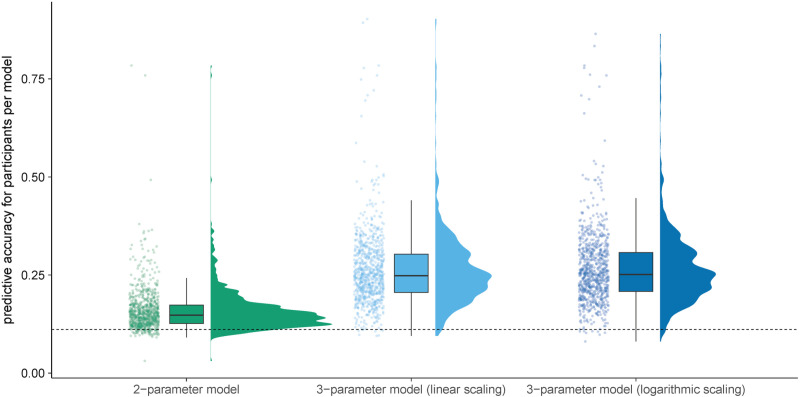
Distribution of predictive accuracies for the modified two-parameter model including a distance parameter, and the two three-parameter models including a cycling-parameter based on either a linear or logarithmic gap distance. Note. The distribution for each parameter is represented through three plots: a density plot (in arbitrary units), a boxplot, and a scatter plot. The bold line in the boxplot represents the median. The whiskers of the boxplot are limited to the IQR * 1.50. The jitter in the scatter plot along the *x*-axis was introduced to make all data points visible. The dashed line at *x* = 1/9 indicates chance performance in predicting the next number in a sequence.

In the next step, we excluded the initial two-parameter model from further analysis as it was surpassed by the two-parameter model that replaced the side-switching parameter with a distance parameter. The three-parameter models were therefore built on the improved version of the two-parameter model as reflected in equation 3 and 4. The predictive probability for all participants regarding each of the three remaining models can be seen in Fig 3. This graph shows the increase in predictive accuracy achieved through the cycling parameter. To provide a comprehensive data presentation, raincloud plots were used for this and all other variable distribution visualizations [43].

Predictive accuracies of the three-parameter model employing a logarithmic scaling of the gap (*M* = 26.48%, *SD* = 9.31%) and the three-parameter model using linear scaling of the gap (*M* = 26.14%, *SD* = 9.29%) were better than for the two-parameter model with the distance parameter. When comparing the three-parameter model with logarithmic scaling of the gap and the two-parameter model, we found that the logarithmic cycling model predicted the subsequent number in a sequence with higher accuracy for 95.06% of the participants. Moreover, there was a clear mean improvement in predictive accuracy when switching from the two-parameter model to the three-parameter model with a logarithmic scaling of the gap, *t*(829) = 42.71, *p* < .001, *d* = 1.25, $\log_{10}BF=\;207.53$. Comparing the three-parameter model with the linear cycling of the gap with the modified two-parameter model, we found that for 94.34% of the participants the linear cycling model allowed for a better prediction of the next number in a sequence. This resulted in a significant increase in mean predictive accuracy, *t*(829) = 40.99, *p* < .001, *d* = 1.22, $\log_{10}BF=\;197.50$. Thus, both the three-parameter model wi*t*h the logarithmic scaling of the gap and the three-parameter model with the linear scaling of the gap outperformed the two-parameter model. These findings suggest that the addition of a cycling parameter to the model enhances its ability to predict choices in an RNG task. Taken together, the results are well in line with H1, which posits that adding a distance and cycling parameter would improve the model’s fit.

When comparing the two three-parameter models with each other, it becomes apparent that the logarithmic cycling model achieved a higher predictive accuracy for 69.76% of the participants. Additionally, mean predictive accuracy was better under the logarithmic model compared to the linear three-parameter model, *t*(829) = 10.24, *p* < .001, *d* = 0.04, $\log_{10}BF=\;19.96$. These findings suggest the presence of a cycling bias, wherein there is a higher likelihood of selecting a number if it has not been chosen recently. Even though the model with the logarithmic scaling of the gap showed a better fit, it should be noted that the absolute difference in predictive accuracy between the two three-parameter models was only relatively small.

Based on these results, we dropped the three-parameter model that assumed a linear scaling of the gap for the cycling parameter from further analyses and relied on the model with a logarithmic scaling of the gap. However, we also retained the two-parameter model with the distance parameter in our subsequent analyses since we aimed to evaluate the effectiveness of the three-parameter model with a logarithmic scaling of the gap in capturing systematic patterns exhibited by humans. In the following analyses, we therefore compared the three-parameter model against the two-parameter model including the generalized distance parameter instead of the original side-switching parameter.

### Simulation of model-driven data

To assess how well our model can account for systematic patterns in human behavior in RNG tasks, we simulated model-generated sequences for the modified two-parameter model using the distance parameter and the three-parameter model using a logarithmic cycling of the gap. We conducted an assessment of both models’ ability to account for biases observed in human behavior in RNG tasks. Using our model and based on the parameter values (the posterior mean) fitted for each model and each participant, we simulated 20 virtual participants with the same parameter values and the same sequence length (= 200). The first number in the simulated sequences was always generated at random and with equal probability from the set of numbers from 1 to 9 as all models need at least one starting number to predict subsequent numbers. This resulted in 4,000 simulated numbers per participant and per model. We also created 20 permuted sequences for each participant to obtain random-like sequences composed of the same numbers as the original sequences (following the procedure proposed by Yousif et al. [6]). The permuted sequences were utilized as a benchmark for comparison, considering that the model-generated sequences ought to be more akin to human-generated sequences than random permutations of the same sequences.

Next, we calculated four measures of randomness across all sequences, including original human-generated, model-generated, and randomly permuted sequences. Our aim was to evaluate the efficacy of the two models in approximating human-generated sequences. In the same data set, the measures we employed were already shown to be sensitive to systematic behavior exhibited by humans [24]. The first measure was the phi index for block size 4 [3,31], a measure of interleaved repetitions. This measure indicates whether there are too many or too few repetitions when comparing the first and last number of blocks of size 4. For this metric, negative values denote insufficient repetitions, whereas positive values indicate an excess of repetitions. The second measure was block entropy for blocks of size 3 [44,45], which is a measure that indicates whether there is an inequality in the frequency of blocks of responses with length 3 in a sequence. The value 0 indicates that only one block of numbers was used throughout; higher values indicate a more even distribution of blocks. The third measure was the coupon score [1], which calculates the average time taken for all numbers to occur at least once in a sequence. Lastly, we calculated the mean gap score [3], indicating the average distance between identical numbers in a sequence.

**Fig 4:**
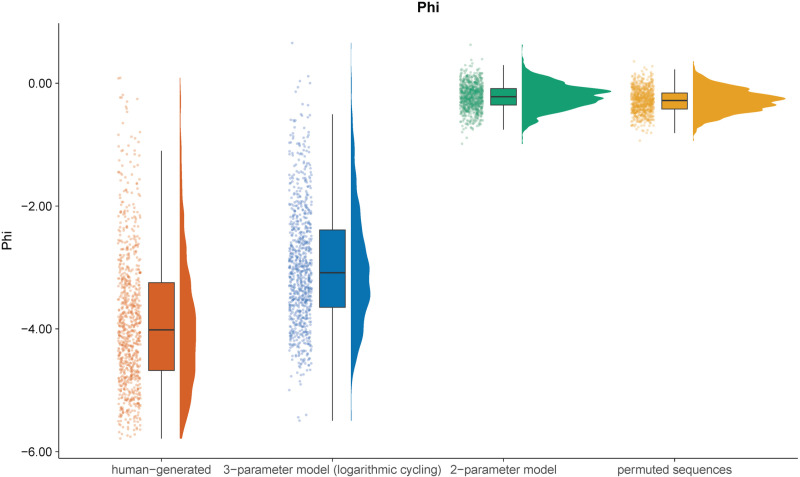
Distribution of the Phi Index over sequences generated by human participants, the three-parameter model with a logarithmic scaling of the gap, the two-parameter model with a distance parameter and random permutations of human-generated sequences. Note. This plot shows the distribution of the phi index computed over different sources of sequences: the human-generated sequences from a RNG task, sequences generated through the three-parameter model with the logarithmic scaling of the gap to the last occurrence of a number, the two-parameter model with a distance instead of a side-switching parameter and sequences that were random permutations of the original-human-generated sequences. The distribution for each source is represented in three plots: a density plot (in arbitrary units), a boxplot, and a scatter plot where the variation along the *x*-axis was introduced to allow better representation of the data points. The bold line in the boxplot represents the median. The whiskers of the boxplot are limited to the IQR * 1.50.

After the computation of the four randomness measures across all human-generated, model-generated, and permuted sequences, we computed for each participant the mean over the randomness measures over the respective participant’s model-generated sequences as well as over the same participant’s permuted sequences. This way, we obtained a mean model prediction for each model per participant for all four randomness measures as well as the same prediction for the randomly permuted sequences belonging to the same participant. If the models could account for the variance in the randomness measures under investigation, we would expect the mean model predictions for a randomness measure to be closer to the observed randomness measures of the participants than the randomness measures computed over the randomly permuted sequences. The distributions of the phi index from all data sources can be found in Fig 4. The distributions of the remaining randomness measures can be found in the Appendix. From a visual inspection, we see that the distribution of the phi index computed over human-generated sequences (*M* = -3.86, *SD* = 1.11) can be best approximated by the sequences generated by the three-parameter model with the logarithmic scaling of the gap for the cycling parameter (*M* = -2.98, *SD* = 0.97). In comparison, the distribution of the phi index computed over sequences generated by the two-parameter model (*M* = -0.22, *SD* = 0.21) is no closer to the distribution of the human-generated sequences than the distribution of the randomly permuted sequences (*M* = -.28, *SD* = 0.19). This indicates that the two-parameter model without the cycling parameter cannot account for the lack of repetitions humans show over moderately long gaps (here between the first and last number of blocks of size 4), as can be seen by the negative phi index values in the human-generated sequences. This is not surprising given that the two-parameter model describes the decision for a number in a sequence only as a function of the number selected immediately before. In contrast, the cycling parameter describes this decision as a result of the complete prior choice history which explains why the three-parameter model can better account for systematic patterns between numbers that are not immediate neighbors.

For the sake of brevity, a full description of the distribution of the remaining three randomness measures for data from different sources is provided in the Appendix. The general pattern was similar for all randomness measures: the approximation of human-generated sequences concerning different measures of randomness was better for the three-parameter model with the logarithmic scaling of the gap for the cycling parameter than for the two-parameter model without a cycling parameter.

To assess the extent of disparity between the sequences that were formed by humans, by the competing models, or by randomly permuting human sequences, we adopted the classification methodology utilized by Angelike and Musch [24]. In doing so, we performed non-parametric bootstrapping (*n* = 1000) to compute logistic regression models employing the randomness measure as the independent variable, and the data source as the dependent variable. This process was repeated for every combination of the human-generated sequences with the other sources (using a three-parameter model with logarithmic cycling, a two-parameter model with the distance parameter, and permuted sequences). For every bootstrapping iteration, we trained the logistic regression model by drawing 1660 random samples with replacement from the computed randomness measures. This sample size was chosen to equal twice the human sample size as we had to pair each human-generated sequence with a sequence from one of the other sources. The correct classification rate for every bootstrapping iteration was established through prediction of the source of generation of the out-of-bag data, which was not part of model training. Thus, we acquired 1000 estimates of the correct classification rate, enabling us to calculate the average and the 2.5th and 97.5th empirical confidence intervals (as presented in square brackets). This methodology was reiterated for each measure of randomness. Correct classification rates of 50% indicate that a randomness measure could discriminate between human-generated and model-generated sequences only at chance level and thus, that the model-generated sequences very closely mimicked human-generated sequences. All results from this analysis can be found in Table 2.

**Table 2:** Rate of correct classifications into human-generated and model-generated sequences based on the Phi-Index, Block Entropy, the Coupon Score, and the Mean Gap score.

Measure	Model		
	Random permutation of original sequences	Two-parameter model with distance parameter	Three-parameter model (logarithmic scaling)
Phi-Index	99.05% [98.39%, 99.67%]	99.02% [98.37%, 99.67%]	67.63% [64.32%, 70.90%]
Block Entropy	92.60% [90.92%, 94.16%]	74.54% [71.19%, 77.78%]	71.57% [67.66%, 75.44%]
Coupon Score	96.41% [95.28%, 97.63%]	95.40% [93.94%, 96.74%]	54.26% [46.61%, 59.77%]
Mean Gap Score	94.68% [93.08%, 96.22%]	92.98% [91.15%, 94.73%]	54.78% [46.10%, 66.51%]

*Note.* Values of 50% indicate that it is not possible to distinguish between human-generated and model-generated sequences of numbers based on standard measures of randomness. Values in square brackets are 95% confidence intervals.

The analysis of the phi index reveals that the disparity between human-generated and three-parameter model-generated sequences was smaller than that between human-generated and two-parameter model-generated sequences, as well as that between human-generated and randomly permuted sequences. Specifically, comparing human-generated sequences with sequences generated with the three-parameter-model using the phi index resulted in a correct classification rate of 67.63% [64.32%, 70.90%] in correctly identifying the generating source. Distinguishing between human-generated sequences and sequences generated with the two-parameter-model was possible with a correct classification rate of 99.02% [98.37%, 99.67%], and distinguishing between human-generated sequences and sequences generated by permuting human-generated sequences was possible with a correct classification rate of 99.05% [98.39%, 99.67%]. The difference in the phi index between the human-generated sequences and the sequences generated by the two-parameter model was significant, as was the difference in the phi index between the human-generated and the permuted human-generated sequences. Taken together, the three-parameter model provided an improvement over the two-parameter model without a cycling parameter or no model at all (randomly permuted sequences). Nevertheless, a distinct dissimilarity was still noticed between the sequences produced by the three-parameter model and the ones created by humans, indicating that the three-parameter model did not approximate human-generated sequences perfectly. The findings obtained with three additional randomness measures (see Appendix) bolster this assertion: the three-parameter model was more successful in approximating organized structures manifested by humans in RNG tasks than the two-parameter model lacking a cycling parameter. The block entropy measure provided the only exception to this rule because regarding this measure, the three-parameter model was only marginally superior to the two-parameter model. These outcomes are well in accordance with H2 as model-driven sequences generated with the three-parameter model exhibited closer similarity to human-generated sequences than to randomly re-ordered human-generated sequences.

### Parameter estimates

For the analysis of parameter estimates, we only used the results of the three-parameter model with the logarithmic scaling of the gap to the last occurrence of a number as all metrics of model fit as well as model-driven simulations indicate that this model provided the best approximation to the biases underlying human RNG. First, we investigated the hyperparameters of the model representing the mean and the standard deviation of the individual parameters. Bayesian credible 95% intervals are provided by the values within square brackets. Participants showed on average a tendency to avoid repetitions (*M* = -0.41 [-0.53, -0.29], *SD* = 1.48 [1.38, 1.58]); however, the standard deviation of this parameter was large. It appears that despite the general trend to avoid repetitions, some individuals showed an opposite tendency and thus, an increased chance of repetitions. The distance parameter was on average negative (*M *= -0.32 [-0.36, -0.27], *SD *= 0.69 [0.65, 0.73], indicating a tendency to choose adjacent pairs (e.g., choosing a 9 after an 8). However, there were some participants that showed the opposite tendency and avoided adjacent numbers. The cycling parameter was positive (*M* = 0.77 [0.74, 0.79], *SD* = 0.37 [0.35, 0.40]), indicating a clear tendency to cycle too fast through all available numbers; the probability of choosing a number increased the longer it was not used. For all three parameters, 95% credible intervals for the hyperparameters representing the means of the individual parameters did not contain the value 0. This finding supports the interpretation that all three parameters can account for a substantial part of human behavior.

**Fig 5:**
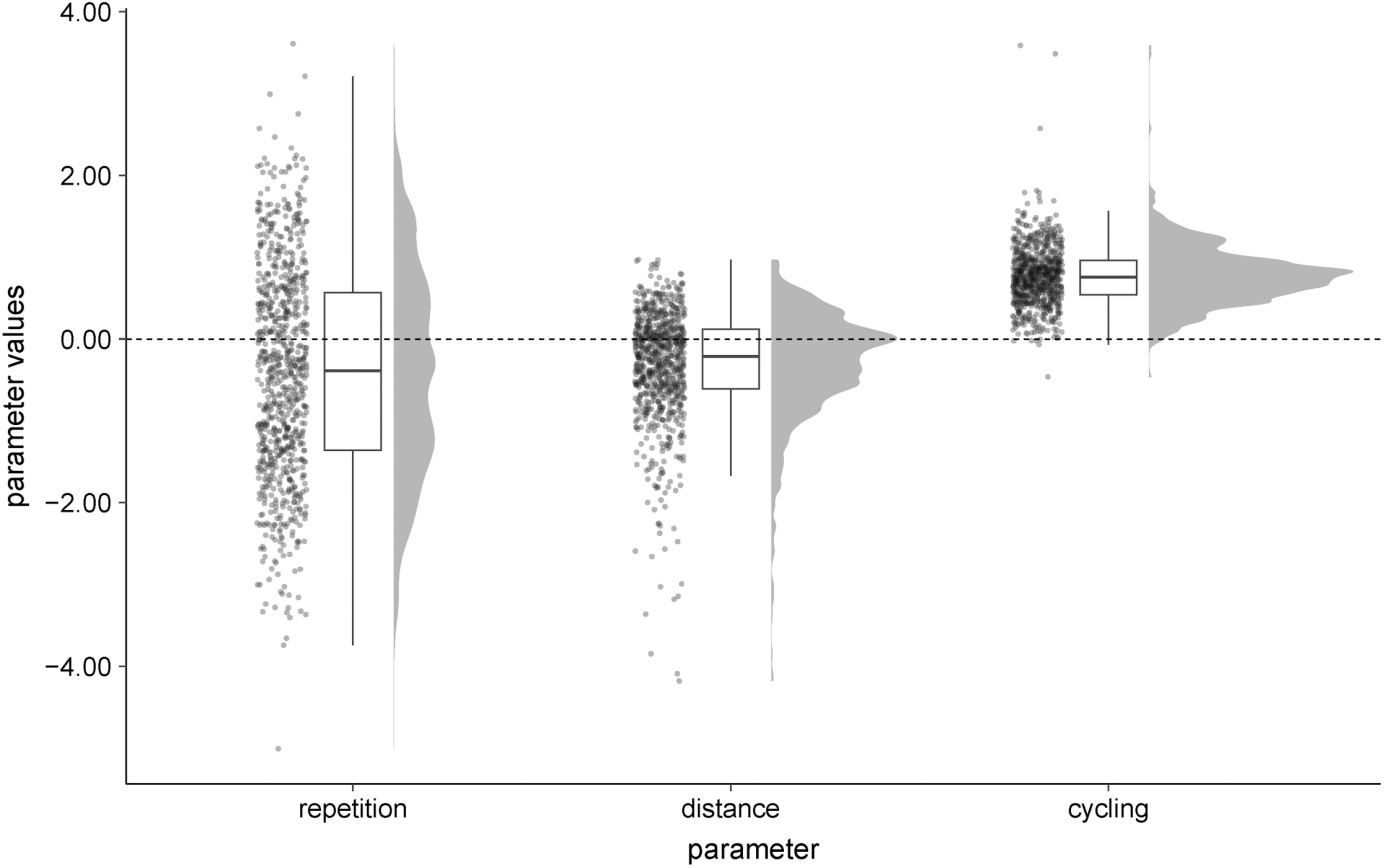
Distribution of the individual repetition, distance, and cycling parameter estimates for the three-parameter model with logarithmic scaling of the gap. Note. The distribution for each parameter is represented in three plots: a density plot (in arbitrary units), a boxplot, and a scatter plot where the variation along the *x*-axis was introduced to allow better representation of the data points. The bold line in the boxplot represents the median. The whiskers of the boxplot are limited to the IQR * 1.50. The dashed line at *y* = 0.00 indicates the absence of a bias.

We also computed all three parameters for each participant by taking the mean over the posterior simulations of the respective participant. The distributions of the individual repetition, distance, and cycling parameters are shown in Fig 5.

We observed some slight differences between men and women regarding the cycling and the repetition parameter. Women showed a somewhat stronger cycling tendency (*M* = 0.80, *SD* = 0.37) than men (*M* = 0.73, *SD* = 0.35), *t*(827) = 3.02, *p* = .003, *d* = 0.21, BF= 6.71. Women also showed, on average, a stronger repetition avoidance (*M* = -0.52, *SD* = 1.25) than men (*M* = -0.29, *SD* = 1.32), *t*(819.29) = 2.54, *p* = .011, *d* = 0.18, BF= 1.82. However, these differences were relatively small and did not change *t*he general pattern of a positive cycling and a negative repetition parameter. Potential causes for the observed differences might be of interest for future research.

### Parameter recovery and correlation

**Fig 6:**
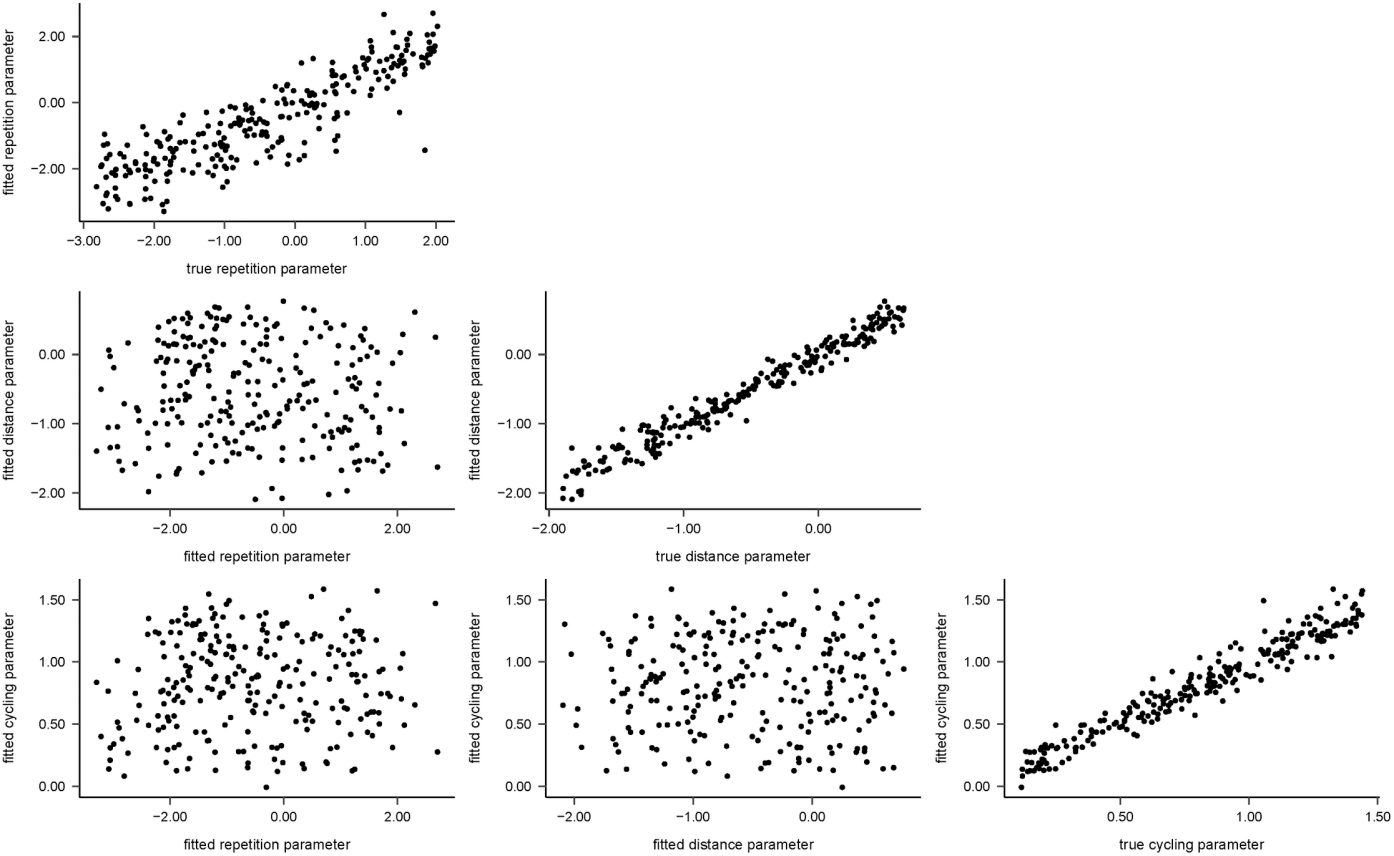
Parameter recovery and parameter correlations for the three-parameter model with the logarithmic scaling of the gap. Note. The diagonal scatter plots represent the parameter recovery results (correlation between true and fitted parameters). The plots to the left of the diagonal represent correlations between different parameters.

Finally, we assessed the reliability of the obtained model estimates from the preceding section in estimating the true parameter values. To this end, we generated 250 number sequences of length 200, comprising numbers 1–9, using the three-parameter model with logarithmic gap scaling, to mimic human-generated sequences. The parameter range for the simulation was established by using the 2.5th and 97.5th percentiles of the individual-level parameter estimates from the human-generated sequences as the lower and upper bounds, respectively. Values within this range were simulated using a uniform distribution. The model was then fitted to the simulated sequences. The fitted parameter values of the simulated sequences were correlated with the true parameter values used for the simulation, avoiding any subjective evaluations. We examined the correlation between the fitted parameter values of various parameters to make sure that they measure different biases in human RNG. The results are shown in Fig 6. It is evident that all three parameters could be retrieved, as evidenced by the robust linear relationship between the true and fitted parameters traced on the scatter plot matrix’s diagonal. This means that the model allowed us to reliably gauge the generating parameters of the sequences. We did not detect any apparent correlation between the various fitted parameter values on the left of the diagonal, suggesting that all parameters could be estimated independently.

## Discussion

We have enhanced Yousif et al.’s computational model [6] by substituting the side-switching parameter with a distance parameter that is less dependent on procedural details of the employed RNG task. Additionally, we added a cycling parameter to account for the phenomenon that humans tend to cycle through all available numbers too quickly. Our findings indicate that a distance parameter is more effective in explaining human behavior in an RNG task than a side-switching parameter. Adding a cycling parameter improved the model fit further. Simulations using the fitted parameters revealed that according to various measures of randomness, our three-parameter model provided an improved approximation to the characteristics of human-generated random sequences. Furthermore, we found that model parameters could reliably be recovered in a simulation, and a lack of correlations between the parameters suggested that all model parameters could be estimated reliably and independently.

Replacing the side-switching parameter in the initial Yousif et al. [6] model with a generalized distance parameter improved the model’s fit to human-generated sequences. Our findings are in line with the findings by Towse [12] who showed that particularly those numbers adjacent or nearby to the previously generated number are selected with increased probability. The distance parameter offers the advantage of being independent of the particular RNG task’s format, as it can be tailored precisely to it. In the present study, the sequences generated by humans were obtained in an RNG task that required the selection of numbers within a 3x3 matrix. The utilization of Euclidean distances between numbers within this two-dimensional space unequivocally enhanced the compatibility of the model to the human-generated sequences. This divulges the significance of incorporating parameters of the RNG task into the modelling process.

The inclusion of the cycling parameter appears to be the most beneficial addition to the model proposed by Yousif et al. [6]. This can be deduced from the distribution of parameter estimates of the three-parameter model with the logarithmic scaling of the gap: the hyperparameter representing the mean of all individual cycling parameters is more than two standard deviations above the point of 0 and the 95% Bayesian credible interval of this parameter does not include 0. The repetition and the distance parameters make a considerably smaller contribution to the performance of the three-parameter model. The cycling parameter can account for more complex behavior as it is influenced by an individual’s entire prior choice history. In contrast, a repetition, side-switching, or distance parameter only reflects the immediately preceding choice and therefore cannot capture the same level of behavioral complexity. This interpretation aligns with prior research, as the inclination to quickly cycle through all feasible numbers among human participants has been frequently observed and probed [1–3,24]. Simulations showed that our expanded model is capable of closely approximating human behavior in RNG tasks. However, despite providing a close approximation to the distribution of the phi index, the coupon, and the mean gap score, the model could not fully mimic the distribution of block entropy scores. Nevertheless, it is noteworthy that the approximation was consistently superior to not applying a model, as evidenced by the distribution of randomness measures computed for randomly permuted human-generated sequences.

With the only exception of block entropy, the three-parameter model with a logarithmic scaling of the gap yielded better approximations to human-generated sequences than the two-parameter model. In order to achieve an even better fit to human behavior, further research is necessary. Yousif et al. [6] reported evidence supporting the suitability of their model by applying repetition and side-switching parameters in various RNG task paradigms. Similar future investigations will likely profit from adding a distance and a cycling parameter.

However, a cycling parameter alone cannot sufficiently account for all human behavior in RNG tasks because on average, the distance parameter assumed negative values, implying participants picked numbers that were close to previously generated ones. This aligns with Towse’s prior research [12]. Participants, on average, tended to avoid repetitions, which previous research has identified as a defining feature of human RNG [11–13]. Nonetheless, it is noteworthy that although a general tendency to avoid repetitions was observed, the variability in this parameter was high, with some participants exhibiting positive parameter values. This finding suggests that some participants do not show an aversion to repetition when other biases like the tendency to cycle through all available numbers too quickly are also taken into account.

When implementing a computational model such as the one used in the present study, it is necessary to formalize all assumptions about human RNG as model equations prior to analysis. This theory-driven approach goes beyond previous research evaluating bias in human RNG tasks. Most studies of human RNG focus on calculating various measures of randomness for the sequences generated by participants. These measures are then often combined using principal component analysis. Researchers have used this approach extensively [1–3,46]. However, measures of randomness can only reflect certain statistical patterns demonstrated by humans in RNG tasks; they cannot, by themselves, be interpreted as latent variables underlying human performance in RNG [47]. A model-based approach, in contrast, allows to directly and jointly quantify various human biases in RNG tasks, and model parameters are always easily interpreted as specified by the model equations.

The computational model described in this paper can be used in various areas of psychological research that examines RNG performance. For example, it can be used to compare the randomization performance of various clinical groups, such as schizophrenic patients [2,48,49], or to explore the impact of age on RNG performance [50–52]. Other applications include testing the impact of auditory distraction [53], manipulating production speed [12], or evaluating the influence of sleep deprivation on RNG performance [54].

## Conclusion

In the present work, we have expanded upon the computational model introduced by Yousif et al. [6] by incorporating two additional parameters: a distance parameter, a more task-independent variant of the original side-switching parameter, and a cycling parameter, which can account for the propensity of many humans to cycle rapidly through all possible numbers in an RNG task. Including both parameters significantly improved the fit of the model and resulted in a better approximation of human behavior in RNG tasks. The cycling parameter is capable of taking into account not only the impact of the last pick on the selection of the subsequent pick in a sequence, but also the impact of an individual’s entire previous selection history. The present promising results suggest that a cycling parameter ought to be integrated into any potential endeavor to assess or simulate human biases in RNG tasks.

## Appendix

### Simulation of model-driven data (continued)

**Fig A1:**
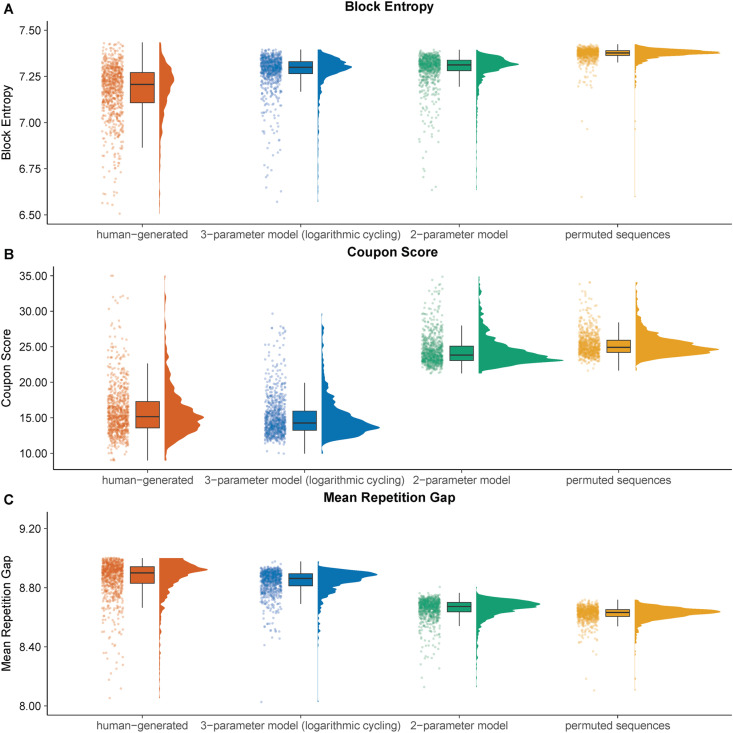
Distribution of randomness measures for human-generated, model-generated, and randomly permuted human-generated sequences. *Note.* This plot shows the distribution of the block entropy for blocks of size 3, the coupon score, and the mean gap score computed over different sources of sequences: sequences generated by humans in a RNG task, sequences generated by the three-parameter model with the logarithmic scaling of the gap to the last occurrence of a number, the two-parameter model with a distance instead of a side-switching parameter, and sequences that were random permutations of the original-human-generated sequences. The distribution for each source is represented in three plots: a density plot (in arbitrary units), a boxplot, and a scatter plot where the variation along the *x*-axis was introduced to allow better representation of the data points. The bold line in the boxplot represents the median. The whiskers of the boxplot are limited to the IQR * 1.50. Block entropy was computed over blocks of size 3 as these block sizes were found to discriminate best between human-generated and random sequences in Angelike and Musch [23]. Long tails of the distributions had to be excluded for the sake of clarity of visualization (there were 40 values below 6.50 and above 7.50 for block entropy, 20 values above 35 for the coupon score, and eight values below 8 and above 9.25 for the mean gap score).

Fig A1 shows the distributions of three measures of randomness (block entropy for blocks of size 3, coupon score, and mean gap score) for data that were generated by humans, by the three-parameter model with a logarithmic scaling of the gap, by the two-parameter model with a distance instead of a side-switching parameter, and by randomly permuting human-generated sequences. As can be seen, the distribution of block entropy scores is least well approximated by the three- and the two-parameter model, though the approximation appears to be an improvement to the distribution of block entropy scores for randomly permuted sequences (human-generated: *M* = 7.12, *SD* = 0.38; three-parameter model: *M* = 7.27, *SD* = 0.18; two-parameter model: *M* = 7.28, *SD* = 0.15; permuted: *M* = 7.37, *SD* = 0.04). The three-parameter model also provided the closest approximation to the distribution of the coupon score (human-generated: *M* = 16.00, *SD* = 4.15, three-parameter model: *M* = 15.36, *SD* = 6.59, two-parameter model: *M* = 24.69, *SD* = 4.56, permuted: *M* = 25.47, *SD* = 2.83). The same was true for the mean repetition gap score (human-generated: *M* = 8.86, *SD* = 0.22, three-parameter model: *M* = 8.83, *SD* = 0.19, two-parameter model: *M* = 8.65, *SD* = 0.13, permuted: *M* = 8.62, *SD* = 0.06).

Regarding block entropy scores, both models produced sequences that more closely mimicked human-generated sequences than permuted sequences (see Table 2). However, due to the overlapping confidence intervals, the data do not show a clear superiority of the three-parameter model over the two-parameter model. Regarding the coupon score, the approximation of the human-generated sequences through the three-parameter model was significantly better than the approximation with the two-parameter model. A similar result could be obtained for the mean gap score. Taken together, our results indicate that the three-parameter model produced sequences for which the distribution of the randomness measures under investigation mimicked the distribution of the randomness measures for human-generated sequences better than both, the sequences produced with the two-parameter model with a distance parameter and the sequences generated by randomly permuting human-generated sequences.
